# The Cure of the "Incurable"

**Published:** 1901-05-18

**Authors:** 


### Hospital Clinics and Medical Progress.

### THE CURE OF THE "INCURABLE."

In a lecture which many, no doubt, will think might well have been headed "never despair," Dr. Charles Bell Taylor[1] describes a series of cases, illustrating the fact that now and again well-directed treatment is successful in restoring sight in cases of apparently incurable blindness—cases which have already undergone much treatment at the hands of careful men, and have been dismissed with the assurance that nothing more is possible.

The cases comprise patients who have recovered after many years of apparently hopeless blindness, and from the effects of neuro-retinitis, iritis with scleroderma, hyalitis, detached retina, atrophy of the optic disc, and the results of destructive inflammatory and degenerative changes.

As to the remedies outside the usual routine which were employed, Dr. Taylor would place electricity first; mercury in large doses, either alone or in combination with other drugs, second; derivatives third; and lastly persevering treatment on such lines together with such operative proceedings as may be indicated "so long as there is the least chance of benefit, or any eye left to be operated on."

Of galvanism he speaks very highly, although he offers no explanation of its good effects beyond the statement that electricity has "a potent influence upon all living protoplasm, a restorative power for which there is no substitute," and acts as a "stimulant, tonic, and catalytic agent." The list of cases in which he has found benefit from its use "includes cases of hysteria, spinal amaurosis, amblyopia, amblyopia exanopsia, blindness or imperfect sight, the result of anæmia or simple atrophy of the disc, atrophy following on typhoid fever and other exhausting diseases, lead-poisoning, and the abuse of alcohol, tobacco, and some other poisons."

In regard to the use of mercury he protests against the notion that mercury, in its non-corrosive forms, is a very dangerous poison, even when salivation is produced; and he reminds us that in the old times a pint or more of saliva was exacted as a daily tribute from the inmates of lock hospitals; and he goes on to mention various cases in which, after all hope had been given up even by eminent men, restoration of sight followed the free and sometimes the prolonged employment of mercury. These were mostly cases of irido-choroiditis followed by effusion into the vitreous humour, and in some mercury had already been given but only in small doses.

Dr. Taylor gives mercury in the form of blue pill, ointment, vapour, and sub-conjunctional injection of the cyanide of mercury. Pilocarpine in half-grain doses, or in smaller doses hypodermically is an immense help, and another useful adjunct is elaterine, which causes profuse watery evacuations, and is especially helpful in cases of detachment of the retina.

Under the heading of derivation Dr. Taylor includes blood-letting (which he does *con amore*, believing that "thick, treacly, diseased blood is better let out"), and sinapisms, blisters, setons, issues, ice-bags, hot-water bags, &c., including the actual cautery. He seems to regard these things as means by which to "distract the attention of the nervous system." "If a vital organ is threatened with extinction or serious damage by inflammation we can save it by establishing another inflammation in a less vital organ near at hand; the attention of the nervous system is distracted; nature cannot support the two inflammations—as one waxes the other will wane, as one grows the other will wither." Here, then, we see the use of setons, issues, and other counter-irritants. As Shakespeare tells us, "one fire burns out another's burning, one pain is lessened by another's anguish"; and as Dr. Taylor tells us, "if one principle in the practice of our profession is more certain than another it is that one pus-forming centre, one inflammatory zone, will dry up another. Nature, I repeat, cannot support the two, and the way to kill one is to foster the other." Dear old Nature! The operative measures undertaken by Dr. Taylor in these condemned cases seem to have been chiefly measures which other surgeons would probably have undertaken could they have brought themselves to believe that there was any hope. And here we think that Dr. Taylor's experiences do teach us a lesson, namely, as to the healing effects of time and rest. The closure of the pupil which so often accompanies or follows diseases by which the deeper structures of the eye have appeared to be disorganised does at least give rest, and here and there it may happen, as seems to have been the case with some of the patients mentioned by Dr. Taylor, that under the influence of prolonged rest the vitreous becomes sufficiently transparent and the retina sufficiently active to give an opening for venturesome and speculative surgery. It is well now and again to review our verdicts, and any surgeon who will carefully and periodically go over the "condemned cases" which exist in numbers in every town—not cases of eyes only, but of spines, and joints, and all kinds of nervous ailments—he will now and again be rewarded by curing the "incurable," and winning fame.

[1] Lancet, April 27.

